# Sickle cell retinopathy in pediatric patients: exploring correlations and predictive factors

**DOI:** 10.1007/s00417-025-06946-y

**Published:** 2025-08-23

**Authors:** Nuno Rodrigues Alves, Catarina Barão, Patrícia Barros Silva, Inês Ludovico, Lívio Costa, Eduardo Silva, Rita Anjos, Rita Serras Pereira

**Affiliations:** 1 Department of Ophthalmology, Unidade Local de Saúde de São José, Lisbon, Portugal; 2 NOVA Medical School, Faculty of Medical Sciences of Lisbon, Lisbon, Portugal; 3 Integrated Responsibility Center for Pediatric Ophthalmology, Hospital Dona Estefânia, Lisbon, Portugal

**Keywords:** Data correlation, Pediatric age, Predictive factors, Sickle cell disease, Sickle cell retinopathy

## Abstract

**Purpose:**

To analyze clinical, laboratory, and Doppler vascular parameters in pediatric sickle cell disease (SCD) patients and identify correlations and predictive factors for sickle cell retinopathy (SCR) and proliferative SCR (PSR).

**Methods:**

A retrospective study included pediatric SCD patients screened for SCR between December 2023 and August 2024. Systemic, transcranial-cervical Doppler, and ophthalmologic evaluations were performed. Correlation analyses explored relationships between clinical, laboratory, and ophthalmologic parameters and SCR. Logistic regression identified predictive factors for SCR and PSR.

**Results:**

We included 172 eyes from 86 pediatric SCD patients (mean age: 11.1 years; 57% male). SCR was diagnosed in 20 patients (23.3%), including 15 with non-proliferative (NPSR) and 5 with PSR. SCR correlated significantly with higher vaso-occlusive crises (VOC) frequency (ρ = 0.379, *p* < 0.001), lower fetal hemoglobin (HbF) (ρ = -0.363, *p* = 0.001), older age (*r* = 0.295, *p* = 0.006), and glucose-6-phosphate dehydrogenase (G6PD) deficiency (ρ = 0.428, *p* < 0.001). Doppler evaluations showed reduced velocities associated with SCR (*p* < 0.05). Logistic regression identified G6PD deficiency (OR = 8.34, *p* = 0.014), increased VOC (OR = 2.22, *p* = 0.011), older age (OR = 1.26, *p* = 0.04), and lower HbF (OR = 0.89, *p* = 0.047) as predictors of SCR. An age cut-off of 11.5 years yielded 65% sensitivity and 64% specificity.

For PSR, significant correlations included SC genotype (*r* = 0.728, *p* < 0.001) and higher hemoglobin (Hb) (*r* = 0.518, *p* = 0.019). Lower hydroxyurea doses were linked to PSR (*r* = -0.548, *p* = 0.012). Hb levels predicted PSR, with a 9.2 g/dL cut-off (80% sensitivity, 86% specificity).

**Conclusion:**

Early detection of SCR is crucial in pediatric SCD patients. Key risk factors include older age (cut-off 11.5 years), higher VOC frequency, G6PD deficiency, and lower HbF for SCR, and SC genotype, reduced hydroxyurea doses, and higher Hb (cut-off 9.2 g/dL) for PSR. Regular ophthalmologic screening and targeted management may help prevent vision loss and improve clinical outcomes.

## Key messages

*****What is known?***:**

- Sickle cell retinopathy (SCR) is the most common and severe ocular complication in sickle cell disease, but data on pediatric cases remain limited, with no consensus on screening timing, frequency, or the predictive value of clinical markers.
- Identifying high-risk children could refine screening protocols and prevent vision loss, but current guidelines lack evidence-based risk stratification.

*****What is new?***:**

- Older age (cut-off 11.5 years), frequent VOCs, G6PD deficiency, and lower HbF levels are key predictors of SCR, while SC genotype and higher hemoglobin (cut-off 9.2 g/dL) predict PSR.
- Reduced doppler vascular velocities correlate with SCR but lack predictive value, while lower hydroxyurea doses are linked to greater PSR severity
- We propose screening at age 9 for SC disease and 13 for SS disease, with closer monitoring and hydroxyurea optimization for high-risk patients to enhance HbF as a protective factor.

## Introduction

Sickle cell disease (SCD) is the most prevalent hemoglobinopathy worldwide and is recognized as a significant public health concern by the World Health Organization [1, 2]. SCD is an autosomal recessive disorder caused by a single amino acid substitution (βS mutation) in the beta-globin chain leading to the production of hemoglobin S (HbS) [2, 3]. Under certain conditions, HbS undergoes polymerization resulting in the deformation of erythrocytes into a sickle shape. This sickling increases blood viscosity, triggers chronic hemolysis, and leads to microvascular occlusions [4]. These pathological processes result in vasculopathy, inflammation, and progressive ischemic organ damage contributing to various systemic complications [5, 6].

SCD encompasses several genotypes with the most common being homozygous HbS/HbS (SS), heterozygous HbS/HbC (SC), and the thalassemic variants HbS/β0/+ thalassemia (Sβ0/+) [7, 8]. Among the many systemic complications of SCD, ocular manifestations are particularly concerning. The most serious ocular complication in SCD patients is sickle cell retinopathy (SCR) which is the most common and severe ocular manifestation of this condition [9].

SCR is a peripheral retinopathy that can be classified into non-proliferative and proliferative forms [10–12]. In 1971, Goldberg proposed a grading system for SCR that remains widely used [13]. Stages I and II represent non-proliferative sickle cell retinopathy (NPSR), characterized by peripheral arteriolar occlusion and arteriovenous anastomosis at the retinal periphery, respectively. Other classical NPSR findings include salmon patches, black sunbursts, and iridescent spots [3, 4, 13]. Stages III, IV, and V correspond to proliferative sickle cell retinopathy (PSR), involving peripheral neovascularization (sea fan formations), vitreous hemorrhage, and retinal detachment, respectively [3, 13].

Arteriolar occlusion is the most characteristic feature of SCR, typically beginning in the temporal retinal periphery of young patients [14]. With age, there is a progression of occluded arterioles and retinal ischemia leading to peripheral vascular remodeling and retinal neovascularization [15]. Visual acuity is a poor indicator of SCR severity, as many patients maintain good vision until the retinopathy is advanced [16, 17]. In SCR patients, retinal ischemic changes may be asymptomatic and painless, potentially leading to neovascularization and blindness if not detected and treated early [18, 19]. In addition to peripheral SCR, recent studies highlight sickle cell maculopathy as an underdiagnosed, often subclinical complication characterized by focal macular thinning and capillary nonperfusion, especially in the deep plexus, detectable by OCT and OCT angiography [20]. While patients often remain asymptomatic, its presence may signal more extensive retinal ischemia and a higher-risk vascular phenotype.

Therefore, there is a need to identify patients with SCR among asymptomatic individuals with SCD, especially those with PSR, who are most at risk of sight-threatening complications.

The prevalence and severity of SCR, along with other ocular complications, increase with age [1]. Studies suggest that SCR develops in up to 42% of individuals during the second decade of life and may cause acute visual dysfunction in 10–20% of cases [21, 22]. A large retrospective study involving 652 patients aged 10 to 25 years found that 33% had NPR, while 6% had PSR [17].

Identifying pediatric patients at high risk for SCR could refine screening protocols, ultimately safeguarding against vision loss. This study aims to analyze clinical, laboratory, and doppler vascular parameters in pediatric SCD patients and to identify predictive factors for SCR and PSR

## Methods

### Study design and sample population

A retrospective cohort observational study was conducted at the Ophthalmology Pediatric Department of Hospital Dona Estefânia (Unidade Local de Saúde de São José) in Lisbon, Portugal, involving pediatric patients with SCD from the Hematology Pediatric Department who underwent screening for SCR between December 2023 and August 2024. The study adhered to the principles of the 1964 Declaration of Helsinki and received approval from the Hospital’s Ethics Committee. Informed consent was obtained from all individual participants’ legal guardians.

Inclusion criteria were pediatric patients (18 years old or younger) with electrophoretic confirmation of SCD diagnosis, regular follow-up appointments, and completion of ophthalmologic, systemic, and neurovascular evaluations with doppler vascular assessments.

Exclusion criteria included patients with a refractive error greater than 6 diopters of spherical equivalent, intraocular pressure (IOP) exceeding 24 mmHg, history of ocular trauma or previous ocular surgery, and evidence of other comorbidities associated with retinopathy, such as congenital or chronic ocular disorders. Sickle cell trait patients were also excluded.

### Data collection

Data was extracted from individual patient records, encompassing clinical, laboratory and doppler vascular parameters. To ensure consistency and relevance, only patients who had completed ophthalmologic, systemic, and neurovascular assessments within the preceding 12 months were included.

### Clinical parameters

Demographic and clinical data, including age, sex, SCD genotype, number of vaso-occlusive crises (VOC) per year (categorized as 0, 1, 2, 3, or ≥ 4 crises), Glucose-6-Phosphate Dehydrogenase (G6PD) deficiency status and hydroxyurea dosage were collected. Annual VOC frequency and hydroxyurea dosing were calculated based on the 12-month period preceding each patient’s ophthalmologic assessment included in the study.

All patients underwent comprehensive ophthalmologic evaluations, performed by experienced retina specialists, including best-corrected visual acuity (BCVA), slit-lamp assessment, and dilated fundus examination. The presence and severity of SCR were assessed using standardized grading systems and patients were classified into two groups: those without retinopathy and those with SCR, further subdivided to NPSR vs. PSR. In patients with bilateral SCR, the eye with the higher grade in Goldberg classification was considered for the study.

### Laboratory parameters

Laboratory data included hemoglobin (Hb, g/dL), hematocrit (Hct, %), mean corpuscular volume (MCV, fL), reticulocyte percentage (%), total bilirubin (g/dL), lactate dehydrogenase (LDH, IU), HbS (%), fetal hemoglobin (HbF, %), and HbC (%). The most recent laboratory results were used unless the patient had undergone a blood transfusion within the last six months or was experiencing an active vaso-occlusive crisis, in which case the laboratory data before the event were considered.

### Doppler vascular parameters

Transcranial and cervical doppler ultrasound were performed to assess vascular parameters, including Time-Averaged Maximum Mean Velocity (TAMM, considered normal if < 170 cm/s), peak systolic velocity (PSV, cm/s), end-diastolic velocity (EDV, cm/s) of the internal carotid arteries (right and left) and resistance index (RI).

### Imaging

In patients with PSR, and in selected cases of NPSR where uncertainty existed, multimodal retinal imaging was conducted, comprising color fundus photography (CFP) using a Topcon Healthcare device and spectral-domain optical coherence tomography (SD-OCT) with a Spectralis (Heidelberg Engineering) system. Fluorescein angiography (FA) was used for phenotype staging according to the Goldberg classification, when needed.

#### Statistical analysis

Statistical analysis was performed using SPSS Statistics, version 27.0 (IBM^®^ SPSS^®^ Statistics, Armonk, NY, USA). Descriptive statistics for continuous variables were expressed as mean and standard deviation (SD). Categorical variables were described using absolute and relative frequencies. The Shapiro-Wilk test was used to assess the normality of the data. The Mann-Whitney test was employed to compare mean differences between groups for non-normally distributed variables, while the independent t-test was applied to normally distributed data. Chi-squared test was used for categorical variables. Correlation analyses were conducted using Pearson or Spearman correlation coefficients, depending on the distribution of the variables, to explore relationships between clinical, laboratory, and ophthalmologic parameters and the presence of SCR and PSR in pediatric SCD patients. The strength of the correlations was classified as follows: small or weak correlation (0.1 to 0.3), moderate correlation (0.3 to 0.5), and strong correlation (0.5 to 1.0). Negative values indicated an inverse relationship, when an increase in one variable corresponded to a decrease in the other. A backward stepwise multivariable logistic regression analysis was subsequently utilized to identify potential predictive factors associated with SCR and PSR. Receiver operating characteristic (ROC) curves were generated to evaluate the predictive ability of continuous variables identified in the logistic regression analysis for SCR and PSR. The area under the curve (AUC) was calculated to assess the diagnostic accuracy of each variable. Cut-off values were chosen based on each parameter’s best sensitivity and specificity.

Missing data was addressed using an imputation method to ensure the integrity of the analysis. Statistical significance was set at *p* < 0.05.

## Results

### Demographic and clinical characteristics

The study analyzed 172 eyes from 86 pediatric SCD patients. The mean age was 11.14 ± 3.58 years (range: 3–18 years). Forty-nine patients (57%) were male. Most patients (*n* = 77; 89.5%) had the SS genotype, followed by the SC genotype (*n* = 6; 7%) and Sβ0/+ (*n* = 3; 3.5%). Twenty patients (23.3%) were diagnosed with SCR, of which 15 had non-proliferative status, and 5 had PSR.

A detailed description of the demographic, clinical, laboratory, and doppler vascular characteristics of the study population is presented in Table 1.

**Table 1:** Demographic and clinical characteristics

Patient characteristics	SCD (*n* = 86)
Age (years; mean, SD)	11.14 (3.58)
Male (n,%)	49 (57%)
Genotype (n, %)	
SS	77 (89.5%)
Sβ0/+	3 (3.5%)
SC	6 (7%)
VOC/year (n, %)	
0	15 (17.4%)
1	28 (32.6%)
2	17 (19.8%)
3	12 (14%)
4	14 (16,3%)
G6PH deficiency (n,%)	20 (23.3%)
HUR dose (micrograms; mean, SD)	689 (397.1)
Laboratory parameters (mean, SD)	
Hemoglobin (g/dL)	8.8 (1.1)
Hematocrit (%)	24.9 (3.04)
Mean corpuscular volume (fL)	83.1 (11.1)
Reticulocyte (%)	7.63 (4.1)
Total bilirrubin (g/dL)	2.01 (1.34)
Lactate dehydrogenase (IU)	506 (167.4)
HbS (%)	71 (16.8)
HbF (%)	10 (7.7)
HbC (%)^#^	45.9 (1.5)
Doppler Vascular Parameters (mean, SD)	
TAMM < 170 cm/s (n,%)	84 (97.7%)
Right ICA assessment	
PSV (cm/s)	224 (97.4)
EDV (cm/s)	87 (44.3)
Resistance index	0.61 (0.08)
Left ICA assessment	
PSV (cm/s)	214 (81.3)
EDV (cm/s)	83 (40.2)
Resistance index	0.61 (0.1)
Sickle cell retinopathy (n,%)	20 (23.3%)

*EDV* (end−diastolic velocity), *G6PD* (glucose−6−phosphate dehydrogenase), HUR (hydroxyurea), *ICA* (internal carotid artery), *PSV* (peak systolic velocity), *SCD* (sickle cell disease), *SD* (standard deviation), *TAMM* (time−averaged maximum mean velocity), *VOC* (vaso−occlusive crises). #HbC was only detected in six patients

### Correlation analysis

SCR was significantly correlated with a higher frequency of VOCs per year (ρ = 0.379, *p* < 0.001), lower HbF levels (ρ = -0.363, *p* = 0.001), older age (*r* = 0.295, *p* = 0.006), reduced PSV in the right internal carotid artery (ρ = -0.232, *p* = 0.032), and lower EDV in the left internal carotid artery (ρ = -0.235, *p* = 0.029). G6PD deficiency also demonstrated a moderate positive association with SCR (ρ = 0.428, *p* < 0.001). No other factors were statistically significant, as detailed in Table 2.

**Table 2:** Correlation coefficients for factors associated with SCR

	Correlation Coefficient (*r*/ρ)	*p*-value
Age	ρ = 0.295	*p* = 0.006*
Male	ρ = −0.200	*p* = 0.064
Genotype	ρ = 0.091	*p* = 0.406
VOC/year	ρ = 0.379	*p* < 0.001*
G6PD deficiency	ρ = 0.428	*p* < 0.001*
HUR dose (micrograms)	ρ = 0.209	*p* = 0.054
Hemoglobin (g/dL)	*r* = −0.002	*p* = 0.984
Hematocrit (%)	*r* = 0.077	*p* = 0.483
Mean corpuscular volume (fL)	*r* = −0.138	*p* = 0.204
Reticulocyte (%)	*r* = 0.047	*p* = 0.670
Total bilirubin (g/dL)	*r* = 0.149	*p* = 0.172
Lactate dehydrogenase (IU)	*r* = 0.107	*p* = 0.328
HbS (%)	*r* = 0.014	*p* = 0.899
HbF (%)	ρ = −0.363	*p* = 0.001*
Right ICA PSV (cm/s)	ρ = −0.232	*p* = 0.032*
Right ICA EDV (cm/s)	ρ = −0.205	*p* = 0.058
Right ICA Gosling Resistance Index	*r* = 0.019	*p* = 0.865
Left ICA PSV (cm/s)	ρ = −0.180	*p* = 0.098
Left ICA EDV (cm/s)	ρ = −0.235	*p* = 0.029*
Left ICA Resistance Index	*r* = 0.075	*p* = 0.494

*EDV* (end−diastolic velocity), *G6PD* (glucose−6−phosphate dehydrogenase), *HUR* (hydroxyurea), *ICA* (internal carotid artery), *PSV* (peak systolic velocity), *VOC* (vaso−occlusive crises).*p−value < 0.05

### Predictive factors

Backward stepwise multivariate logistic regression analysis identified four key predictive factors for SCR: G6PD deficiency (Odds Ratio (OR) = 8.341, *p* = 0.014), an increased number of VOC per year (OR = 2.216, *p* = 0.011), higher age (OR = 1.262, *p* = 0.04), and lower HbF (OR = 0.885, *p* = 0.047). No other factors were statistically significant The only continuous variable predictor of SCR was age (area under the curve (AUC) = 0.697) with a cut-off of 11.50 years, sensitivity, and specificity for the presence of SCR of 65% and 64%, respectively. HbF (AUC < 0.5) did not confirm to be a good predictive factor isolated

### Subgroup analysis: retinopathy group characterization and correlations

Among the 20 pediatric patients with SCR, 15 (17.4% of the population) had NPSR and 5 (5.8%) had PSR. The mean age for NPSR was 13.47 years (youngest age 8 years for both SS and SC genotypes), while for PSR it was 11.80 years (youngest 10 years for SC genotype vs. 13 years for SS genotype). In terms of the Goldberg classification, most NPSR patients were classified as Stage I or II (73.3% and 26.7%, respectively), whereas all PSR patients were classified as Stage III (100%) (Table 3)

**Table 3:** Retinopathy group characterization

SCR (*n* = 20; 23.3%)	NPSR (*n* = 15; 17.4%)	PSR (*n* = 5; 5.8%)
Age (years; mean; SD)	13.47 (3.09)	11.80 (3.90)
SS (youngest age)	8	13
SC (youngest age)	8	10
Goldberg Classification (n, %)		
I	10 (75%)	-
II	5 (25%)	-
III	-	5 (100%)
Genotype (n, %)		
SS	15 (100%)	2 (40%)
Sthal	0	0
SC	0	3 (60%)

*SCD* (sickle cell disease), *NPSR* (non−proliferative sickle retinopathy), *PSR* (proliferative sickle retinopathy), *SD* (standard deviation)

Hemoglobin levels were significantly higher in PSR patients (mean Hb 9.88 g/dL) compared to those with NPSR (mean Hb 8.47 g/dL) (*p* = 0.019). Additionally, PSR patients had a lower dose of hydroxyurea (400 µg) than NPSR patients (970 µg) (*p* = 0.012), though genotype differences may influence this.

Significant correlations with PSR included SC genotype (*r* = 0.728, *p* < 0.001), and higher Hb levels (*r* = 0.518, *p* = 0.019). However, we acknowledge that only six patients with the HbSC genotype were included, which limits the statistical power and generalizability of genotype-specific conclusions. Despite this limitation, the strong correlation observed aligns with existing evidence.

Lower hydroxyurea doses were also correlated with an increased likelihood of PSR (*r* = −0.548, *p* = 0.012). Among these, only Hb levels demonstrated a strong predictive value for PSR (AUC = 0.82), with a cut-off of 9.2 g/dL providing 80% sensitivity and 86% specificity. In contrast, hydroxyurea dose had a poor predictive value isolated (AUC = 0.187).

## Discussion and conclusion

 The current literature on SCR in children is limited. Our study found an overall prevalence of SCR among pediatric SCD patients screened by an ophthalmologist to be 23.3%. Of these, 17.4% had NPSR and 5.8% had PSR, which aligns with the ranges reported in previous studies (Table 3) [16, 23, 24].

PSR typically develops between the ages of 15 and 19 in SS patients and between 10 and 14 in SC patients, with two-thirds of PSR cases occurring between ages 15 and 29 [25, 26]. In our cohort, the onset of NPSR occurred at similar ages for both the SC and SS genotypes, with the youngest diagnosis being 8 years old. Among the 5 children diagnosed with PSR, 3 had the SC genotype and 2 had the SS genotype. The youngest age at PSR diagnosis was 10 years in SC children and 13 years in SS children. These findings are consistent with previous studies on SCR in children (Table 4) [16, 23, 24].

**Table 4:** Studies reporting prevalence and youngest age at diagnosis of SCR in pediatric patients

Articles	Overall Cohort		SC genotype		SS genotype	
	Prevalence of NPSR (%)	Prevalence of PSR (%)	Youngest NPSR (years)	Youngest PRS (years)	Youngest NPSR (years)	Youngest PRS (years)
Gill and Lam, 2008 [23]	16.7	2.7	7	9	7	16
Rosenberg and Hutcheson, 2011 [24]	16.3	4.3	6	11	6	13
Li, 2019 [16]	11.1	2.3	5	12	6	15
Current study	17.4	5.8	8	10	8	13

There is still no clear consensus on whether children with SCD should undergo routine ophthalmologic screening, the ideal age to start such exams, or how often they should be performed. The literature also varies on whether clinical markers of SCD reliably predict the risk of SCR, making it unclear how they should guide screening guidelines.

To better stratify risk, we analyzed various potential risk factors for SCR in pediatric patients, incorporating demographic, clinical, doppler vascular, and laboratory data, some of which have not been previously reported. Earlier studies, such as those by Gill and Lam [23], Rosenberg and Hutcheson [24], and Li [16], included patients from the 1980 s to 2013. Since then, advances in SCD management, such as routine analytic evaluations, annual transcranial doppler screenings, long-term chronic transfusions, and hydroxyurea use have emerged [27]. Therefore, we sought to assess the impact of these parameters on SCR risk.

We identified several trends for SCR development, including older age (cut-off 11.50 years, sensitivity 65%, specificity 64%), a higher number of VOCs, G6PD deficiency, and lower HbF levels. While lower doppler vascular velocities were correlated with SCR, they were not predictive. Interestingly, both the SC genotype and higher hemoglobin levels (cut-off 9.2 g/dL, sensitivity 80%, specificity 86%) were predictive for the development of PSR. Lower hydroxyurea doses were correlated with PSR but not predictive.

None of the other evaluated risk factors were associated with SCR or PSR development. For previously studied risk factors, these results are not surprising, given the variability of results among studies assessing such in the past [28–31].

Advancing age and a higher frequency of VOCs reflect increased systemic sickling activity, resulting in reduced blood flow and exacerbated retinal ischemia [14, 15]. This can lead to significant remodeling of the peripheral vasculature and stimulate the production of angiogenic factors, ultimately promoting neovascularization [14]. These findings underscore the need for effective management strategies to minimize VOC frequency and severity, emphasizing hydration, pain management, and hydroxyurea use.

The association between G6PD deficiency and SCR has been documented in only one previous study [24]. G6PD deficiency can increase oxidative stress and hemolysis, potentially exacerbating the condition in patients with SCD [32]. The role of oxidative stress in vascular health is significant and may contribute to the pathogenesis of SCR. Future research should aim to elucidate the mechanisms by which G6PD deficiency affects vascular complications in SCD.

Our findings on cervical doppler vascular parameters are intriguing and novel. We observed that patients with SCR exhibited lower peak systolic velocity (PSV) and end-diastolic velocity (EDV). These changes are likely attributable to increased blood viscosity linked to the sickle-shaped cells, particularly when compounded by additional risk factors. This elevated viscosity may lead to microvascular occlusions and progressive ischemic damage to the retina [4]. Consequently, these vascular alterations could serve as potential markers for heightened SCR risk.

Male gender and lower HbF levels have been associated with SCR development in some studies, but results are inconsistent [23, 24, 33]. For instance, Rosenberg and Hutcheson [24] found that male gender correlated with an increased risk of SCR, while Gill and Lam [23], and Li [16] reported no such correlation. Although our study did not achieve statistical significance, it indicates a trend toward increased risk in males.

HbF has consistently been identified as protective against SCR, and our findings align with this [33, 34]. One study demonstrated that the incidence of SCR inversely correlates with HbF levels, showing that levels above 15% are protective during childhood in a large pediatric SCD population [33]. Additionally, Serras-Pereira et al. reported that an HbF cut-off of 3% had sensitivities and specificities of 90% and 75%, respectively, for the presence of PSR [34].

Furthermore, in patients treated with hydroxyurea, pharmacological induction of HbF may provide significant protective benefits against SCR, supporting our results [33]. These factors could either have a protective effect or serve as markers for disease severity, potentially correlating with a higher risk of SCR. However, it is often underused in HbSC patients, who have higher SCR risk but tend to be less anemic. This under-prescription may leave them less protected. In our study, lower hydroxyurea doses were linked to proliferative SCR but not as an independent predictor. This gap in care suggests the need for closer monitoring and better hydroxyurea use to raise HbF and reduce SCR risk.

Interestingly, previous studies have shown that higher hemoglobin levels are associated with PSR severity [28, 35, 36]. Higher hemoglobin levels might indicate a more severe form of SCD, leading to increased complications like sickling, resulting in vasculopathy, inflammation and progressive ischemia and neovascular remodeling.

Several limitations to our study should be considered. First, the retrospective design introduces inherent biases, such as selection bias, and data collection may be affected by inconsistencies in medical records. Second, the study was conducted at a single national tertiary center, which limits how well the results represent the wider population’s genotype distribution. We did not use OCT imaging as screening, which can reveal early retinal changes that precede clinically visible SCR. Although earlier studies using OCT and OCTA to detect early retinal changes in children with SCD often had small sample sizes and limited ability to assess peripheral lesions [37–39], more recent and larger studies support the utility of these imaging modalities for earlier detection and monitoring of disease progression [40]. Although emerging literature suggests that retinal thinning on OCT and vascular changes on OCT angiography can help diagnose retinopathy earlier in children with SCD, these studies often involved small sample sizes and missed peripheral lesions [37–39]Additionally, we did not employ a screening with fluorescein angiography, which has been shown to detect peripheral changes not visible during standard fundoscopic examinations, potentially increasing screening sensitivity [39]. However, fluorescein angiography is invasive and may not be practical or available in all pediatric ophthalmology settings. While advancements in imaging may enhance future screening [41], our methodology reflects standard practices among most ophthalmologists, who often do not use imaging for every patient due to time, cost constraints, and uncertain benefits in this population. For younger children, such as those in our study, the effectiveness of advanced imaging may be limited due to their ability to comply with these procedures.

Ophthalmologic screening for children with SCD is essential to identify asymptomatic SCR, as early treatment can help prevent sight-threatening complications. Current guidelines, including those by Yamn et al. from the American Academy of Pediatrics recommend that pediatric SCD patients undergo a dilated funduscopic examination starting at age 10, although the quality of evidence supporting this recommendation is low [27]. These age-based recommendations are often easier for patients and caregivers to follow than genotype-stratified schedules. Screening should ideally begin at the earliest age when SCR – particularly its proliferative form - may develop, similar to guidelines for retinopathy of prematurity [42]. Based on our study and prior research [16, 23], we propose that screening for PCR should start at age 9 for children with SC disease and at age 13 for those with SS disease, using 11.50 years as a cut-off for SCR development (Table 5). Importantly, our recommendation is intended to supplement, not replace, universal early baseline screening for all children with SCD, since SCR can develop earlier. In our cohort, the youngest age at diagnosis of NPSR was 8 years for both HbSS and HbSC genotypes, underscoring the need for initial early screening even if genotype-stratified ages are used to guide closer follow-up for proliferative disease.

**Table 5:** Screening recommendations for SCR

Author	Age to begin screening	Frequency	Type of screening
American Academy of Pediatrics, 2014 [27]	10 years, especially for SC genotype	Annual -biennial	Dilated funduscopic examination
Babalola and Wambebe, 2001 [43]	10 years	Biennial until age 20	Dilated funduscopic examination, fluorescein angiography if available
Gill and Lam, 2008 [23]	9 years for SC genotype and 13 years for SS and Sβ0/+	Biennial if normal	Dilated funduscopic examination, fluorescein angiography if anormal
Li, 2019 [16]	9 years for SC genotype and 13 years for SS	Annual (especially if NPSR)	Dilated funduscopic examination, fluorescein angiography if anormal

Future longitudinal studies are needed to investigate SCR progression and assess the effectiveness of targeted interventions in reducing its incidence. Additionally, research on non-invasive screening imaging methods may help detect pre-symptomatic changes more effectively compared to current standard practices.

In summary, our study offers groundbreaking insights into the risk factors for SCR in pediatric patients, systematically integrating clinical, laboratory, and doppler vascular parameters in a novel manner. We identified critical indicators for high-risk patients, including lower HbF levels (with a literature cut-off of 3%), reduced hydroxyurea dosages, elevated hemoglobin levels (cut-off of 9.2 g/dL), G6PD deficiency, and a higher frequency of VOCs, all of which are significantly associated with an increased likelihood of SCR development and severity.

We recommend that clinicians prioritize more frequent evaluations for these high-risk pediatric patients and explore strategies to optimize hydroxyurea treatment to enhance HbF levels, which serve as a protective and modifiable factor. By clarifying these relationships, our research contributes to a deeper understanding of the pathophysiology of SCR and supports the creation of targeted screening protocols. These insights are essential for refining clinical management strategies, with the ultimate goal of reducing the incidence of vision loss and improving the quality of life for this vulnerable population.
