# Correction: Sickle cell retinopathy in pediatric patients: exploring correlations and predictive factors

**DOI:** 10.1007/s00417-026-07154-y

**Published:** 2026-02-19

**Authors:** Nuno Rodrigues Alves, Catarina Barão, Patrícia Barros Silva, Inês Ludovico, Lívio Costa, Eduardo Silva, Rita Anjos, Rita Serras Pereira

**Affiliations:** 1 Department of Ophthalmology, Unidade Local de Saúde de São José, Lisbon, Portugal; 2 Faculty of Medical Sciences of Lisbon, NOVA Medical School, Lisbon, Portugal; 3 Integrated Responsibility Center for Pediatric Ophthalmology, Hospital Dona Estefânia, Lisbon, Portugal

**Correction to: Graefe's Archive for Clinical and Experimental Ophthalmology (2025) 263:3237**-**3245**

10.1007/s00417-025-06946-y

In this article, the author list was published incorrectly due to an unintentional omission during the production process.

The original author list omitted the following author(s):

Rita Anjos, Department of Ophthalmology, Unidade Local de Saúde de São José, Lisbon, Portugal; NOVA Medical School, Faculty of Medical Sciences of Lisbon, Lisbon, Portugal.

Rita Serras Pereira, Department of Ophthalmology, Unidade Local de Saúde de São José, Lisbon, Portugal.

The complete and correct author byline is as follows:

Nuno Rodrigues Alves, Catarina Barão, Patrícia Barros Silva, Inês Ludovico, Lívio Costa, Eduardo Silva, Rita Anjos and Rita Serras Pereira.

The original article has been corrected.

